# Delay for cholecystectomy after common bile duct clearance with ERCP is just running after recurrent biliary event

**DOI:** 10.1007/s00464-023-10423-0

**Published:** 2023-09-19

**Authors:** Eric Bergeron, Théo Doyon, Thibaut Manière, Étienne Désilets

**Affiliations:** 1 Department of Surgery, Charles-LeMoyne Hospital, 3120, Boulevard Taschereau, Greenfield Park, QC J4V 2H1 Canada; 2 Department of Gastroenterology, Charles-LeMoyne Hospital, Greenfield Park, QC Canada

**Keywords:** Endoscopic retrograde cholangiopancreatography, Choledocholithiasis, Gallstones, Recurrence, Cholecystectomy

## Abstract

**Background:**

Gallstone disease will affect 15% of the adult population with concomitant common bile duct stone (CBDS) occurring in up to 30%. Endoscopic retrograde cholangiopancreatography (ERCP) is the mainstay of management for removal of CBDS, as cholecystectomy for the prevention of recurrent biliary event (RBE). RBE occurs in up to 47% if cholecystectomy is not done. The goal of this study was to evaluate the timing of occurrence of RBE after common bile duct clearance with ERCP and associated outcomes.

**Methods:**

The records of all patients who underwent ERCP for gallstone disease followed by cholecystectomy, in a single center from 2010 to 2022, were reviewed. All RBE were identified. Actuarial incidence of RBE was built. Patients with and without RBE were compared.

**Results:**

The study population is composed of 529 patients. Mean age was 58.0 (18–95). There were 221 RBE in 151 patients (28.5%), 39/151 (25.8%) having more than one episode. The most frequent RBE was acute cholecystitis (n = 104) followed by recurrent CBDS (n = 95). Median time for first RBE was 34 days. Actuarial incidence of RBE started from 2.5% at 7 days to reach 53.3% at 1 year. Incidence-rate of RBE was 2.9 per 100 person-months. Patients with RBE had significant longer hospitalisation time (11.7 vs 6.4 days; *P* < 0.0001), longer operative time (66 vs 48 min; *P* < 0.0001), longer postoperative stay (2.9 vs 0.9 days; *P* < 0.0001), higher open surgery rate (7.9% vs 1.3%; *P* < 0.0001), and more complicated pathology (23.8% vs 5.8%; *P* < 0.0001) and cholecystitis (64.2% vs 25.9%; *P* < 0.0001) as final diagnoses.

**Conclusions:**

RBE occurred in 28.5% of the subjects at a median time of 34 days, with an incidence of 2.5% as early as 1 week. Cholecystectomy should be done preferably within 7 days after common bile duct clearance in order to prevent RBE and adverse outcomes.

Gallstone disease affects 7–15% of the adult population throughout the life [1, 2]. Prevalence may reach 80% at the age of 90 [3, 4]. Concomitant common bile duct stone (CBDS) occurs in 5–30% of the patients [2, 5–7].

Bile duct stone is the primary cause of acute pancreatitis [8, 9], which can be severe in 20–30% of the acute pancreatitis cases [9]. Overall mortality associated with pancreatitis is 5% [9, 10] and can reach 20–30% in severe cases [10, 11]. Mortality associated with cholangitis may reach more than 10% [12].

Endoscopic retrograde cholangiopancreatography (ERCP) is the mainstay of management for the removal of CBDS [11, 13]. ERCP remains insufficient, since recurrence of CBDS occurs in up to 30% [4, 14], and recurrence of any related biliary events (RBE) in up to 47% of the cases [15, 16] despite clearance of the common bile duct (CBD). While ERCP fails to prevent further RBE [7, 16–18], cholecystectomy does prevent the RBE in majority of the cases [15, 17, 19, 20].

Cholecystectomy is associated with diminished recurrence of cholangitis [9, 21–23], pancreatitis [24–27], or any biliary events after clearance of CBD [7, 16, 18, 28–32]. Guidelines thus recommend cholecystectomy early after common bile duct clearance but for varying time periods from 24 h to 4 weeks [2, 5–7, 12, 18, 20, 22, 32–41].

The goal of this study was to evaluate the timing of recurrent CBDS and other biliary events after clearance with ERCP. This study also aimed to identify the factors associated with this adverse outcome.

## Materials and methods

This is a retrospective study of patients who underwent an endoscopic retrograde cholangiopancreatography (ERCP) for gallstone disease followed by cholecystectomy at Charles-LeMoyne Hospital, Quebec, Canada. Medical records were individually reviewed. Preoperative, operative and postoperative data were collected. This study has been approved by the Charles-LeMoyne Research Center and Charles-LeMoyne Hospital Ethics committee.

This study population is composed of patients aged 18 years or older who underwent ERCP followed by cholecystectomy from July 2010 to June 2022. Cholecystectomy records were extracted from the Operating Room database. ERCP records were extracted from the Endoscopy and Radiology databases. Database records were matched to identify patients who underwent ERCP before surgery. Exclusion criteria were: patients less than 18 years of age, ERCP for reasons other than gallstone disease, severe pancreatitis, cholecystectomy carried out before ERCP.

Information on the following independent variables was collected: age, sex, American Society of Anesthesiology (ASA) score, Charlson Comorbidity index, and initial diagnosis related to the biliary disease.

Each episode of care related with gallstone disease was reviewed. The following data were collected: types of admission (emergency or elective), diagnoses, index investigation or not and reasons to delay interventions, timing of all ERCPs, installation of stent, clearance of CBD or not, number of ERCPs needed for the clearance of CBD, number of ERCPs before surgery, types and recurrence of biliary events (RBE), date of surgery, reason if no index operation, and length of hospital stay for each admission.

The primary outcome variable was recurrence of all biliary events. Groups with or without RBE were compared. Secondary outcome variables were, duration of surgery, conversion or open surgery, surgical complications, postoperative stay, pathological status of the gallbladder and death.

Delay for clearance of CBD was calculated between the first presentation and the first ERCP that showed no residual CBDS. Delay for surgery after clearance was calculated from the ERCP with clearance of CBD and operation. The time of occurrence of RBE after clearance of the CBD was plotted on Kaplan–Meier curves and cholecystectomy was the censored time. Chi-square test or two-tailed Student’s t test were used for analyzing independent variables. For outcome variables, Chi-square test was used for discrete variables and ANOVA for continuous variables. Wilcoxon rank-sum test was used to compare medians. Statistical significance was established at *P* < 0.05.

## Results

During the 12-year-study period (July 2010 to June 2022), 529 patients underwent cholecystectomy after having ERCP for gallstone related biliary disease. Among the studied patients, 57.1% were females (Table 1). Mean age of all the patients was 58.0 years, with 47.6% of them over 60 years, 29.2% over 70 years, and 8.7% over 80 years. Comorbidities were present in 52.2% of the patients, with 25.7% having more than one. Charlson Comorbidity index and distribution of ASA score are presented in Table 1.

**Table 1:** Demographics and preoperative data

	Total	RBE	No-RBE	*P*
Total	529 (100.0)	151 (28.5)	378 (71.5)	
Female	302 (57.1)	93 (61.6)	210 (55.1)	0.1741
Male	227 (42.9)	58 (38.4)	171 (44.9)	
Age	58.0 ± 17.1	56.5 ± 16.4	57.6 ± 17.4	0.6148
18–59	277 (52.3)	89 (58.9)	188 (49.7)	
60+ (vs 18–59)	252 (47.6)	62 (41.0)	190 (50.3)	0.0556
70+ (vs 18–69)	155 (29.2)	37 (24.5)	118 (31.2)	0.1255
80+ (vs 18–79)	46 (8.7)	8 (5.3)	38 (10.0)	0.0886
Comorbidities				
Charlson comorbidity index	2.03 (1.78)	1.79 (1.71)	2.13 (1.80)	0.0527
ASA score	1.99 ± 0.62	1.93 ± 0.62	2.01 ± 0.62	0.1973
I	102 (19.3)	33 (21.8)	69 (18.2)	0.4462
II	333 (62.9)	96 (63.6)	237 (62.7)	
III	92 (17.4)	21 (13.9)	71 (18.8)	
IV	2 (0.4)	1 (0.7)	1 (0.3)	
V	0 (0.0)	0 (0.0)	0 (0.0)	
Initial diagnosis				
CBDS/no pancreatitis/no cholangitis	343 (64.8)	111 (73.5)	232 (61.4)	0.0104*
Cholangitis	110 (20.8)	29 (19.2)	81 (21.4)	
Pancreatitis	66 (12.5)	11 (7.3)	55 (14.5)	
Miscellaneous	10 (1.9)	0 (0.0)	10 (2.6)	
Initial reason to delay cholecystectomy	458 (86.6)	151 (100)	307 (81.2)	
External reference after index ERCP	222 (42.0)	85 (56.2)	137 (36.2)	0.1065
External reference for investigation	182 (34.4)	48 (31.8)	134 (35.4)	
Clearance of CBDS not completed	39 (7.4)	12 (7.9)	27 (7.1)	
Medical reason	8 (1.5)	4 (2.6)	4 (1.0)	
Miscellaneous	7 (1.3)	2 (1.3)	5 (1.3)	
Index cholecystectomy	71 (13.4)	0 (0.0)	71 (18.8)	
Stent during ERCP	239 (45.2)	100 (66.2)	139 (36.8)	< 0.0001*
Clearance achieved	174 (32.9)	82 (54.3)	92 (24.3)	
Clearance not achieved	65 (12.2)	18 (11.9)	47 (12.4)	
No stent during ERCP	290 (54.8)	51 (33.8)	239 (63.2)	
Delay before clearance with ERCP (days)	13.8 ± 25.2	12.8 ± 23.0	15.1 ± 26.1	0.3457
Number of ERCP for clearance				
1	447 (84.5)	129 (85.4)	318 (84.1)	0.8012
2	70 (13.2)	20 (13.2)	50 (13.2)	
3	11 (2.1)	2 (1.3)	9 (2.4)	
4	1 (0.2)	0 (0.0)	1 (0.3)	
Delay between clearance and surgery				
Mean (days)	100.0 ± 170.9	139.3 ± 280.6	84.2 ± 93.2	0.0001*
Median (IQR 25–75) (days) all patients	64 (26–127)	83 (31–161)	58 (19–116)	< 0.0001*
Median (IQR 25–75) (days) excl. index	80 (41–143)	83 (31–161)	78 (43–136)	0.0007*

Results are presented as n (%), mean ± standard error, median (interquartile range) when appropriate

*Statistically significant

Indication for ERCP was common bile duct stone without cholangitis or pancreatitis in 64.8% of the patients, cholangitis in 20.8%, and pancreatitis in 12.5%. A total of 624 ERCP were necessary to achieve initial clearance; one ERCP in 84.5%, two ERCP in 13.2%, three ERCP in 2.1%, and four ERCP in 0.2% of the cases. A stent was installed in 45.2% of the patients after initial presentation (Table 1). During the waiting period for cholecystectomy, 90 supplementary ERCP were carried out.

Cholecystectomy was carried out after a median time of 64 days (IQR 26–127 days) (Table 1). In 71 cases (13.4%), cholecystectomy was done during the same admission. In patients with delayed cholecystectomy, the median time before surgery was 80 days (IQR 41–143 days). There were 308 patients (58.2%) with clearance of CBD with ERCP at the initial admission. After exclusion of patients unfit for index surgery, 222 cases (42.0%) were referred for planned elective cholecystectomy, despite the clearance of the CBD at index admission.

**Fig. 1:**
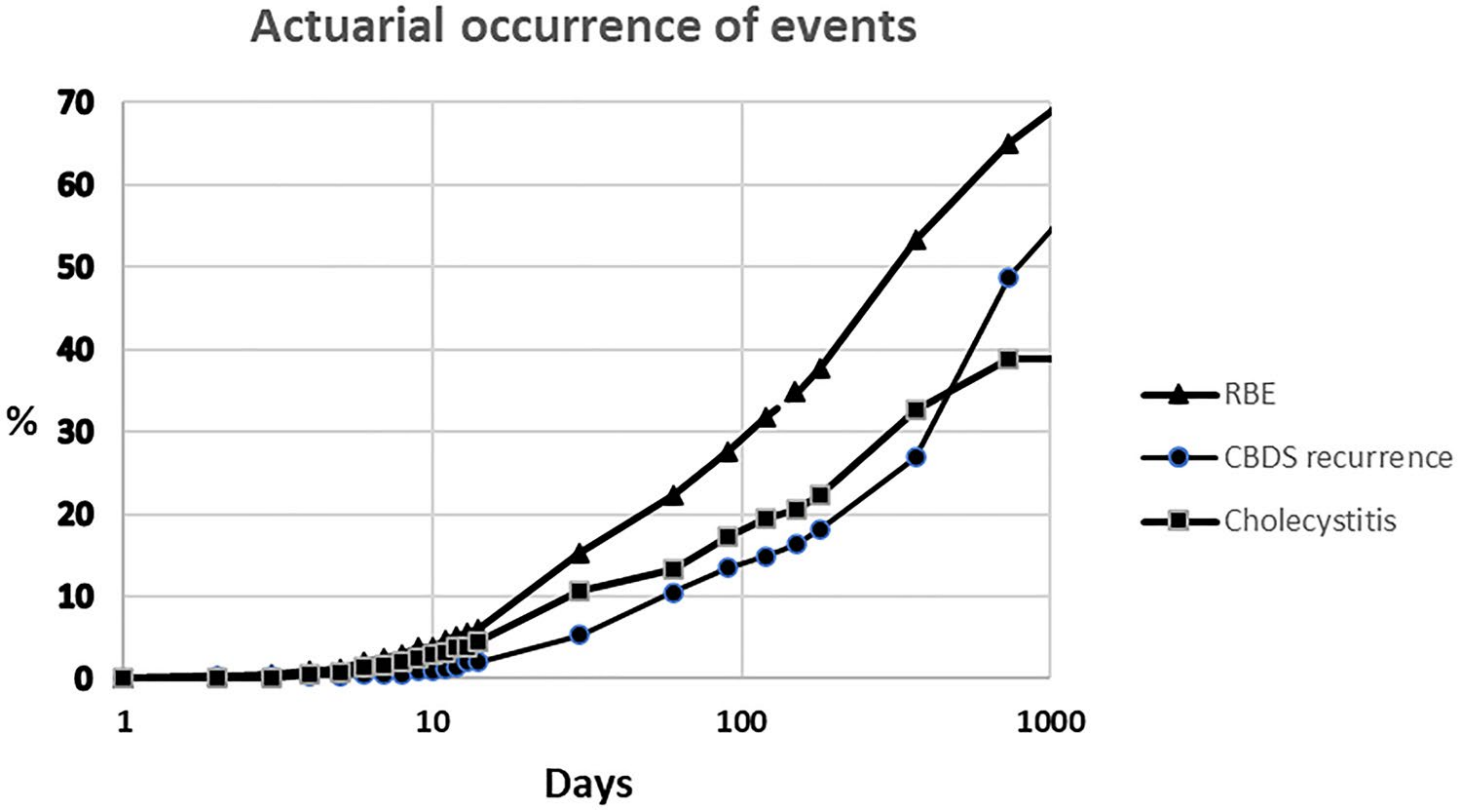
Actuarial occurrence of acute cholecystitis, recurrent CBDS, and all types of RBE after clearance of the common bile duct with ERCP

**Fig. 2:**
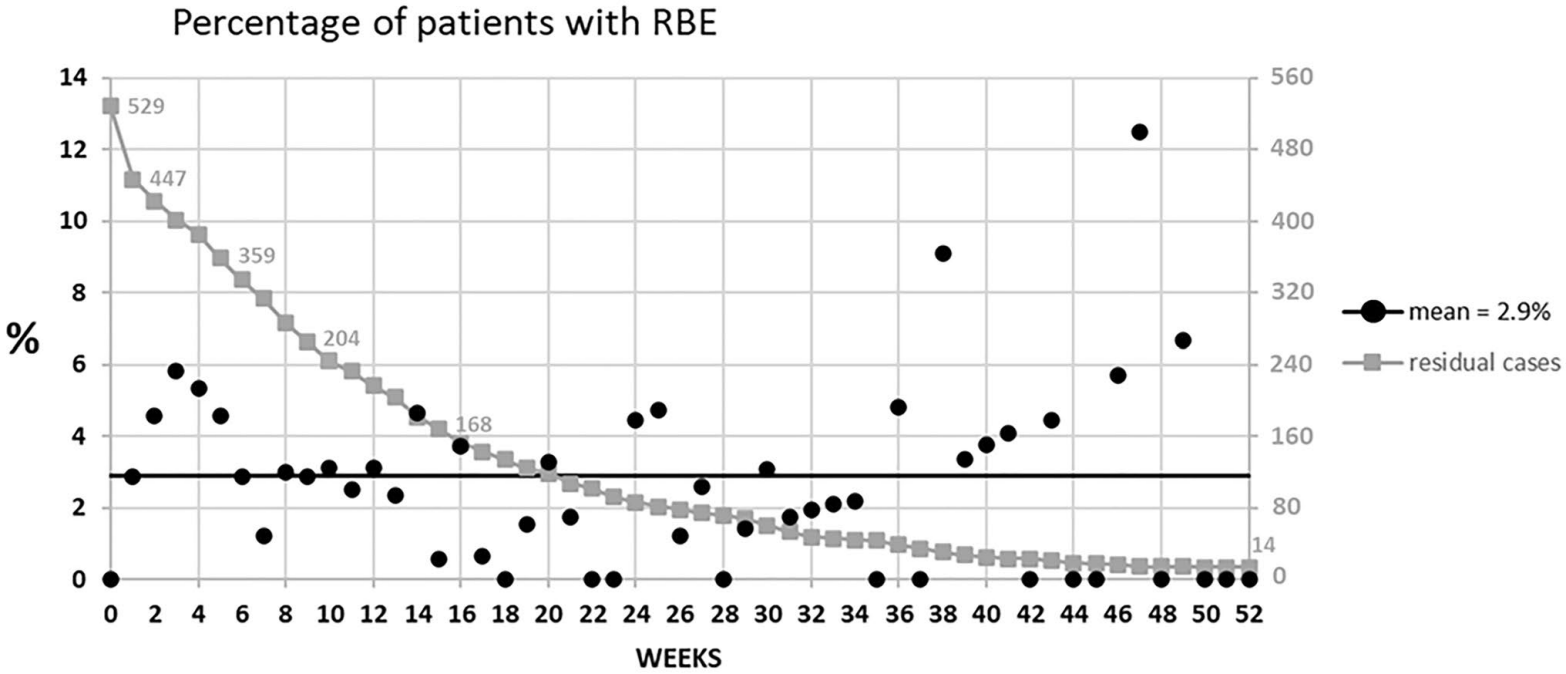
Incidence of recurrent biliary events over time

While awaiting cholecystectomy, there were 221 episodes of RBE in 151 patients (28.5% of the studied patients); 39 patients with RBE (25.8% of those with RBE) had more than one episode. Cholecystitis was the most frequent gallstone related event in 47.0% (Table 2) with the first episode occurring at a median time of 29.5 days (IQR 15–82 days). At least one recurrent episode of common bile duct stone with or without pancreatitis/cholangitis occurred in 69 patients (13.0%), which occurred at a median of 42 days (IQR 25–94 days). In twelve of these patients (16.2%), there was more than one episode related to CBDS. Overall, the first RBE occurred at a median of 34 days (IQR 19–89 days). Figure 1 shows the actuarial occurrence of RBE, recurrent of CBDS and cholecystitis after clearance. Actuarial occurrence of RBE was 2.5% at 7 days up to 53.3% at 1 year. Figure 2 shows the relative incidence of RBE in patients before cholecystectomy. The incidence-rate of RBE was 2.9 cases per 100 person-months.

**Table 2:** Types of recurrent biliary events (RBE)

Type	n (%)
Total^a^	221 (100.0)
Acute cholecystitis	104 (47.0)
Complicated^b^	33 (14.9)
Common bile duct stone	95 (43.0)
No pancreatitis/no cholangitis	65 (29.4)
Cholangitis	22 (9.9)
Pancreatitis	8 (3.6)
Biliary colic	16 (7.2)
ERCP complication	4 (1.8)
Liver abscess	2 (0.9)

^a^In 151 patients with RBE

^b^Perforated, gangrenous or abscessed

Patients with and without RBE are also compared in Table 1. There was no difference in age and gender between groups except for a tendency for more RBE in patients under 60 years of age. Globally, comorbidities and ASA are comparable between groups. RBE was significantly more frequent when initial diagnosis was CBDS with no associated pancreatitis or cholangitis (73.5% vs 61.4%; *P* < 0.05). RBE was significantly more frequent when patients are referred for elective surgery after clearance is achieved with ERCP during the initial admission. RBE were also significantly more frequent when a stent was initially installed. In addition, within the group of patients with a stent, RBE occurred significantly more frequently if clearance was achieved (47.1% vs 27.7%; *P* = 0.0067). The number of ERCP necessary to complete clearance was not significantly different between groups.

The group of patients who suffered RBE had significantly longer hospital stay and postoperative stay (Table 3). They also experienced significantly longer operative time and significantly more open or conversion to open intervention. There was significantly more cholecystitis and complicated pathology in patients who had RBE. There was no difference in surgical complications and CBDS after surgery. Two injuries to the common bile duct occurred, one in each group. Globally, only 10 cases (1.9%) experience recurrence of CBDS after cholecystectomy. Only one death was encountered in a patient with a severe septic shock.

**Table 3:** Outcome variables

	Total	RBE	No-RBE	*P*
n (%)	529 (100.0)	151 (28.5)	378 (71.5)	
Initial length of stay (days)	4.89 ± 5.31	4.98 ± 5.55	4.85 ± 5.22	0.8062
Total length of stay (days)	7.89 ± 8.03	11.7 ± 11.2	6.37 ± 5.65	< 0.0001*
Postoperative stay (days)	1.48 ± 3.14	2.92 ± 4.63	0.90 ± 2.03	< 0.0001*
Failed planned 1-day surgery	83/366 (22.7)	37/78 (50.0)	46/288 (16.0)	< 0.0001*
Duration of surgery (minutes)	53.9 ± 26.9	66.6 ± 34.0	48.8 ± 21.4	< 0.0001*
Conversion/open surgery	17 (3.2)	12 (7.9)	5 (1.3)	< 0.0001*
Surgical complications	26 (4.9)	11 (7.3)	15 (4.0)	0.1110
Final pathologic diagnosis: Cholecystitis	195 (36.9)	97 (64.2)	98 (25.9)	< 0.0001
Complicated	58 (10.9)	36 (23.8)	22 (5.8)	< 0.0001
CBDS recurrence after surgery	10 (1.9)	4 (2.6)	6 (1.6)	0.4057
Death	1 (0.3)	0 (0.0)	1 (0.3)	0.5270

Results are presented as n (%) and mean ± standard error when appropriate

*Statistically significant

## Discussion

Almost half of the patients who undergo ERCP may suffer an episode of recurrent biliary event (RBE) even if clearance of the common bile duct (CBD) is achieved [16, 29]. RBE may be biliary colic, cholecystitis, hepatic abscess, and choledocholithiasis with or without pancreatitis or cholangitis [7, 22, 31]. Specifically, up to 30% of the patients may have recurrent common bile duct stone (CBDS) [14].

In this retrospective study, all types of gallstone related events after ERCP and clearance of CBD are included. The 28.5% occurrence of RBE after endoscopic CBDS extraction in this series correlates with a previously reported incidence of 17% to 60% [4, 12, 15, 19, 22, 25, 29, 31, 42, 43]. In two prospective randomized trials, after a wait-and-see policy, 24% [30] and 47% [29] of the patients presented with RBE, respectively. Schiphorst et al., found a 20% incidence of RBE at median of 22 days [31]. In a national registry study, Sandzen et al., found readmission for RBE within 1 year after acute biliary pancreatitis in, respectively, 62% and 76% of patients without cholecystectomy, depending upon if they had endoscopic sphincterotomy or not [33]. Regarding the cumulative incidence of RBE, it increases rapidly to reach 53.3% in 1 year (Fig. 1). At 1 week, the cumulative incidence was already 2.5% and increased almost linearly thereafter, with an incidence rate of 2.9% per 100 person-months. It means that patients are exposed to a steady risk of RBE while awaiting cholecystectomy. Huang et al. reported a 10.3% cumulative incidence of RBE at 60 days [7] while Cheng et al., reported 18.4% at 1 year [43]. However, these results were drawn from databases, with potential limitations of missing episodes, contrary to the present study in which all records were individually reviewed.

Just considering the recurrence of CBDS, its incidence is reported to occur between 9 and 30% [14, 30, 44–46]. In a nationwide population-based study, Park et al. found a first-time recurrence of CBDS in 11.3% of the patients [45]. In a prospective randomized study, Lau et al. found 18% of recurrent CBDS in patients who did not undergo cholecystectomy after 36 months of follow-up [30]. Kawaji et al. reported the occurrence of CBDS after ERCP in 12.4% of patients, and multiple recurrence in 21.5% of patients, who suffered a first recurrence of CBDS [44]. The present study shows, after exclusion of patients who underwent cholecystectomy during initial admission, that 15.5% of the patients had episodes of recurrent CBDS. Also, considering only CBDS related episodes, 12 out of 74 patients (16.2%) had a second recurrence. Our results thus correlate with the reported iterative incidence of CDBS of 16% [47] and 23% [45], respectively. Multiple recurrences must thus be an important concern.

The high incidence of acute cholecystitis in this study is surprising (Table 2). We wonder if it is because we searched it more closely. In addition, patients with final pathology report of acute cholecystitis after elective cholecystectomy are even not included, since surgery is the censored time. Episodes of intercurrent acute cholecystitis are thus real RBE. Actually, acute cholecystitis after ERCP gained few attention [48] since most studies have mainly focused on the identification of CBDS recurrence [46]. We, however, underline that cholecystitis is an important biliary event to be considered.

Cholecystectomy has been proven to prevent occurrence of RBE or readmissions after removal of choledocholithiasis [3, 7, 16, 20, 29–31]. Patients who underwent cholecystectomy after endoscopic extraction of CBDS showed a decreased hazard of RBE [21]. A Cochrane review by McAlister et al. demonstrated more RBE in wait-and-see strategy, with 35% of patients needed “rescue” cholecystectomy [16]. The gallbladder left in situ is an independent risk factor of RBE [44, 49, 50] and cholecystectomy provides a protective effect on the recurrence of CBDS [7, 30, 31, 43]. In fact, cholecystectomy has proven to be the strongest protective factor against readmissions [49, 51].

Cholecystectomy, either early or delayed, thus remains the best protector against RBE [29, 30, 51], approaching 90% of protection [7]. In addition, reviews showed that RBE is definitively lower if cholecystectomy is carried out early vs delayed (2–10% vs 24–47%) [13, 15, 29–31]. While a better reduction in the risk of RBE is achieved the sooner cholecystectomy is carried out [12, 42, 52], the definition of “early” cholecystectomy after endoscopic treatment of choledocholithiasis remains variable [1, 28, 53]. This is reflected in guidelines recommending early cholecystectomy after periods varying from 24 h to 4 weeks [2, 5–7, 12, 18, 20, 22, 32–41]. A recent study using a nationwide readmission database found that the lowest risk of readmission after ERCP for choledocholithiasis was index cholecystectomy [54]. A 5% risk of RBE has been demonstrated in PUNCHO trial within 24 h after pancreatitis [12].

There are several reasons for the decision to delay cholecystectomy including limited resources and operating room time, economic restraints, surgeon’s comfort and decision, and patient’s choice. [2, 31, 49, 54]. Another one is the belief and demonstration that cholecystectomy is more difficult after ERCP and endoscopic sphincterotomy [55–58]. However, cholecystectomy remains technically as difficult or even become more laborious if carried out later [15, 29, 30, 53, 55, 56, 58, 59], thus making sense to undertake early intervention [56, 59]. One limitation of this study is the difficulty to extract the reason to delay surgery after ERCP, i.e. surgeon’s choice vs patient’s preference. In the majority of cases of index ERCP, we figure out that it is the decision of the surgeon since the patients are admitted in the surgery service. During the follow-up, it remains also difficult to differentiate between the surgeon’s and the patient’s decision. However, the delay in planned elective surgery clearly and mainly relies on the lack of elective operating room availability.

Comorbidity is certainly another factor that is taken into consideration to delay cholecystectomy. Expectant management or delayed cholecystectomy is significantly more frequent in elderly and patients with more comorbidities [21, 23, 42, 60]. Aziz et al. showed that comorbidities are significantly associated with readmissions [60]. In the present study, elderly and patients with higher comorbidity score are not significantly associated with an increased incidence of RBE (Table 1). Yet, index cholecystectomy after ERCP have been demonstrated to be secure, with fewer or at least no increased risk of complications [12, 13, 25–27, 42, 49, 51], along with the prevention of RBE [3, 7, 16, 20, 21, 29–31, 43, 44, 49–51]. Age and comorbidity should not be an obstacle or a deterrent to proceed to early cholecystectomy or even at the index admission after the endoscopic removal of CBDS, except when risk is prohibitive [4].

The median delay that reaches 80 days before elective cholecystectomy is mainly imputable to the lack of resources and elective operating time. Analysis of the waiting period remains limited since information on the exact factors or reasons of delays are difficult to collate from the records retrospectively. In addition, considering emergency admission leading to “earlier” cholecystectomy in cases of RBE, the delay before elective would have been increased furthermore. Delays to elective surgery are definitively too long accounting for median times for occurrence of RBE or CBDS of respectively 34 and 42 days. Amazingly, half of the episodes thus occur before elective cholecystectomy. In a similar study, Schiphorst et al. found the same situation, with a median time between ERCP and elective cholecystectomy of 7 weeks while the median time was 22 days for the occurrence of RBE [31]. Limited resources are clearly identified as a significant problem [2, 31, 54] impeding implementation of guidelines [49].

The operative time in the group of patients who experienced RBE was significantly longer. Operative time may represent a surrogate of the difficulty of operation [53]. Operative time is higher after a previous ERCP [55], in emergency setting [28] and increases according to the number of episodes of cholecystitis [57]. Although the goal of this study was not to evaluate the relationship between cholecystitis and ERCP, its high incidence deserves to be considered as a contributing risk in the difficulty of delayed operation.

Unlike the majority of studies evaluating the occurrence and risk of RBE and complications in delayed cholecystectomy [7, 13, 17, 19, 21, 24, 28, 35], the present study rather examined the outcome in patients suffering RBE independently of the timing of surgery. Even if the occurrence of surgical complications did not reach statistical significance between groups, open cholecystectomy, either planned or converted, was significantly more frequent in patients who had RBE (Table 3). Schiphorst et al. also compared patients who had RBE and found an incidence of postoperative complications of 24% vs 11% in patients without RBE before cholecystectomy [31]. Open cholecystectomy is not necessarily associated with surgical complications, and should not be considered as such, but certainly has a consequence on the length of hospital stay and recovery period. Higher rates of conversion have been associated with longer time between ERCP and cholecystectomy [55, 59, 61]. Tracy et al. also found a higher conversion rate despite the lack of any difference in surgical complications, emphasizing however, the impact on the postoperative length of stay [61].

Earlier cholecystectomy after ERCP and common bile duct extraction of stones has largely been evaluated. Risk of RBE is well demonstrated in delayed cholecystectomy [4, 12, 14, 15, 19, 22, 25, 29–31, 33, 42–47]. On the other hand, early cholecystectomy was not associated with increased surgical complications [4, 15, 27, 28, 31, 53] or conversion to open surgery [19, 25, 27, 28, 31, 53, 55, 62]. Some studies even demonstrated association with a better surgical outcome in early cholecystectomy [13, 29, 30, 59, 61]. Consequently, guidelines have been elaborated but with varying time frames [2, 5–7, 12, 18, 20, 22, 32–41] reflecting the lack of evidence of optimal timing of cholecystectomy [28, 34] from the prospective randomized controlled trials [18, 19].

UK guidelines [20], American Society Gastrointestinal Endoscopy (ASGE) [32] and European Society of Gastrointestinal Endoscopy (ESGE) [2] recommend cholecystectomy within 2 weeks after removal of common duct stone. We must, however, underline that the actuarial incidence of RBE is already 6.1% at 14 days with 15.5% of episodes occurring with 14 days. Other studies report 26.7% [19] and 31.3% [35] of RBE occurring within 2 weeks. Considering that index cholecystectomy has proven to be safe in patients fit for surgery [3, 7, 12, 13, 16, 20, 21, 25–27, 29–31, 42–44, 49–51], it should be the best option. In our study, no case of RBE occurred during index admission. Despite the guidelines to undergo index cholecystectomy [5, 6, 36–39], the compliance is recently reported to be less than 50% [39, 52, 54, 60], and even in decline, for unknown and complex reasons [42, 52, 54, 60].

For patients discharged without cholecystectomy or referred on external basis, with or without ERCP and clearance, the investigation must be rapidly actualized [12, 42, 52]. Some studies have shown a decrease in CBDS recurrence and cholangitis using a prophylactic stent [63, 64]. However, a significant increase in RBE in patients who had a stent, even if clearance was achieved, was found in this study (Table 1), but this includes an important proportion of cholecystitis. We emphasize that the goal of this study was not to specifically address the contribution of stenting. Moreover, conclusions about prophylactic stenting also remain limited since the reason to leave a stent is difficult to obtain from a retrospective point of view (choice of endoscopist, edema, bleeding, prophylactic). While stenting may be an effective step for definitive clearance of CBDS [65], cholecystectomy soon after clearance remains necessary and should not wait for the removal of the stent [64].

A recent systematic review and meta-analysis concluded that surgery should be coordinated within preferably 72 h [62, 66]. The major impediment to proceed early is the lack of elective time, as reported by others [18, 49, 54]. Even if surgeons believe that surgery should be delayed, almost half of the elective cholecystectomy procedures are carried out after 3 months [31, 49]. So, performing cholecystectomy during the same admission, using an acute care surgery model, would considerably decrease the elective burden [22, 67]. There is no local expertise for laparoscopic common bile duct exploration (LCBDE). Developing this evolving modality could help to further alleviate the number of interventions, complications of ERCP, hospital stays, costs, and burden of elective interventions [68–71]. The problem of operating room availability for acute care surgery would, however, remain. A coordinated fast track investigation and treatment, with a dedicated team and institutional commitment towards fulfillment of guidelines remains mandatory [72]. Also, continuous and rigorous evaluation in the quality of care and compliance with guidelines should be implemented [34, 67, 73, 74].

The single-centre retrospective design of this study presents some strengths and limitations. Inasmuch as all charts were individually reviewed, missing data should be minimal, unlike the data taken from registries. In this way, all RBE have probably been identified, since readmissions and ERCP reports are readily available. Only patients who underwent cholecystectomy were included, making it not possible to draw conclusions on patients without surgery. Patients who had intraoperative cholangiogram are also not included since they did not have ERCP before cholecystectomy. Conclusions cannot be drawn for highly severe comorbid patients with ASA ≥ 4. Moreover, very elderly patients (80+) remain underrepresented. In addition, the reason for the decision not to operate, nor the precise moment when the cholecystectomy was exactly planned, were difficult to extract from the hospital records. Hence, patients with a decision not to operate are not included and conclusions cannot be drawn for this specific group. Patients may be missed if they were seen in another center for either RBE, surgery, or postoperative complications. It was also not possible to identify patients who could have died or suffered major complications (related or not with gallstone disease) impeding surgery. Notwithstanding these limitations of this study and its retrospective design, the total number of patients and distribution between groups, renders the analysis and conclusions, in our opinion, highly valuable.

In conclusion, this study highlighted the following after clearance of CBDS with ERCP:

- RBE occurred in 28.5% of patients.
- RBE recurred in 25.8% of patients after a first episode of RBE.
- Actuarial occurrence of RBE start from 2.5% at 7 days to reach 53.3% in 1 year.
- Incidence rate of RBE remains steady at 2.9 per 100 person-months before cholecystectomy.
- Comorbidity and elderly are not significantly associated with the occurrence of RBE.
- RBE is associated with a significantly higher risk of difficult operation, conversion and surgical complications.

Our results reinforce that cholecystectomy should be carried out, should the patient be fit for surgery, the earliest after ERCP for gallstone disease and clearance of CBD. Ideally, cholecystectomy should be carried during the same admission, or within the very first few days, but definitively not beyond seven days. Otherwise, any delay just significantly runs after recurrent biliary event.
